# A Review of Recent Legal Decisions Affecting Physicians, Dentists, Druggists and the Public Health

**Published:** 1899-09

**Authors:** 


A Review of Recent Legal Decisions Affecting Physicians, Dentists, Druggists and the Public Health. Together with a Brief for the Prosecution of Unlicensed Practitioners of Medicine, Dentistry or Pharmacy, with a Paper upon Manslaughter, Christian Science and the Law and Other Matter. By W. A. Purrington, of the New York Bar, Counsel of the Dental Society of the State of New York; Lecturer of Medical and Dental Jurisprudence in the New York College of Dentistry, and one of the collaborators in A System of Legal Medicine, by Allan McLane Hamilton and others, etc. Duodecimo, pp. 105. New York: E. B. Treat & Co. 1899.

The cases and decisions presented in this book are well reviewed by Mr. W. A. Purrington of the New York Bar, who is now identified as a writer and lecturer of reputation in medical jurisprudence. The increase in the number of legal cases, specifically affecting these professions and of legal cases demanding for their elucidation, expert knowledge in these vocations has become so great, that their collections in a volume by themselves is fully justified.

When, however, in addition to their being thus made of ready access, they are by so qualified a person as Mr. Purrington divested of dry extraneous matter and made as reliable as interesting, there would appear nothing omitted to fit them for the purpose intended.

Brevity is attained without sacrificing particular or important matter. Criticism is to the point and instructive, references are exact and complete, the arrangement simple.

It covers the field of medicine, dentistry, pharmacy and public health; it should and undoubtedly will find its way to the library of the lawyer as well as those of the professions concerned. For the reasons specified, many will feel it fills a decided want.

The publisher for his enterprise, and the author for the quality of his work, has placed us under obligations. W. H. H.

